# Punch Card Programmable Microfluidics

**DOI:** 10.1371/journal.pone.0115993

**Published:** 2015-03-04

**Authors:** George Korir, Manu Prakash

**Affiliations:** Department of Bioengineering, Stanford University, 443 Via Ortega, Stanford, California, 94305, United States of America; University of Illinois at Chicago, UNITED STATES

## Abstract

Small volume fluid handling in single and multiphase microfluidics provides a promising strategy for efficient bio-chemical assays, low-cost point-of-care diagnostics and new approaches to scientific discoveries. However multiple barriers exist towards low-cost field deployment of programmable microfluidics. Incorporating multiple pumps, mixers and discrete valve based control of nanoliter fluids and droplets in an integrated, programmable manner without additional required external components has remained elusive. Combining the idea of punch card programming with arbitrary fluid control, here we describe a self-contained, hand-crank powered, multiplex and robust programmable microfluidic platform. A paper tape encodes information as a series of punched holes. A mechanical reader/actuator reads these paper tapes and correspondingly executes operations onto a microfluidic chip coupled to the platform in a plug-and-play fashion. Enabled by the complexity of codes that can be represented by a series of holes in punched paper tapes, we demonstrate independent control of 15 on-chip pumps with enhanced mixing, normally-closed valves and a novel on-demand impact-based droplet generator. We demonstrate robustness of operation by encoding a string of characters representing the word “PUNCHCARD MICROFLUIDICS” using the droplet generator. Multiplexing is demonstrated by implementing an example colorimetric water quality assays for pH, ammonia, nitrite and nitrate content in different water samples. With its portable and robust design, low cost and ease-of-use, we envision punch card programmable microfluidics will bring complex control of microfluidic chips into field-based applications in low-resource settings and in the hands of children around the world.

## Significance Statement

The capacity to implement complex robust multiplex assays in resource poor settings devoid of skilled personnel, power sources and supportive infrastructure can revolutionize difficult to execute applications in global health, environmental monitoring and forensics, anywhere around the world. Combining microfluidics with programming using paper punch card tapes, here we present a novel integrated general-purpose fluidic platform to address specific challenges for resource-poor settings. Powered manually by a hand-crank, our device incorporates a single-layer microfluidic chip in a plug-and-play fashion and is programmed by a paper tape with punched holes as discrete instructions. In addition to the above-mentioned applications, we aspire to enable children to have access to “programmable chemistry kits” in science education settings globally.

The use of microfluidic technology, where small volumes of fluids are manipulated in carrying out miniaturized laboratory assays, has drawn considerable attention owing to inherent advantages that include minimized reagent consumption, miniaturized reaction volumes and the potential to yield robust and rapid results [1]. Application of microfluidics for robust multiplex diagnostic tests in extremely low-resource settings holds great promise but remains currently unfulfilled due to a variety of challenging factors including absence of electricity, lack of refrigeration for reagent storage, unavailable calibration services for devices over time, challenging operating conditions such as fluctuating temperatures and lack of skilled personnel [2]. There is an especially urgent need for multiplexed tests either to diagnose a disease caused by multiple agents, aid in the differential diagnosis of diseases that clinically present similarly or cases of co-morbidities due to a high disease burden in developing countries[2]. Therefore a successful implementation in these settings requires surmounting the above-mentioned challenges, using a platform that is completely self-contained and modular in nature, coupled with access to stable reagents that can be easily replenished.

The capability for performing robust and inexpensive assays that are easy to replicate has applications beyond medical diagnostics. When coupled to the capacity to easily manipulate fluids in a programmable fashion that is easy to implement and run, one can envision new applications in science education settings worldwide. Hands-on introduction of chemistry and biology for school children can instill a life-long passion for science [3]. Although many current scientists admit to having been inspired by open-ended explorations utilizing chemistry kits widely available several decades ago, safety concerns and expensive reagents have made this exploration currently unavailable. Low-cost self-sufficient microfluidic technologies with enclosed chemicals and small-volume reagent reservoirs could potentially provide a wide-ranging solution to the problems mentioned above.

With the implementation of pneumatic micro-valves, it is now possible to run thousands of assays in parallel on the same microfluidic chip [4, 5]. Although significant progress has been made in development and manufacturing of complex microfluidic chips, current external control systems that are often required remain bulky and expensive [2,6]. A few applications for microfluidic devices in educational settings have been explored before, primarily focused on micro-fabrication techniques [7] while others have focused on applying existing platforms to teach principles of fluid dynamics [8]. However a gap still exists for a robust platform that can carry out complex multiplex assays yet being easy to program and use as desired.

To address some of the challenges brought about by specific constrains in low-resource settings, several novel approaches have been implemented, including a finger-actuated microfluidic pump device [9] and battery-powered implementation of pneumatic valves using solenoids [10]. While promising, such approaches are either limited in range of fluid manipulation or still utilize external solenoid valves that can significantly increase device costs and depend on electrical or battery power-source. Dipsticks and lateral flow assays and in general “paper microfluidics” have found greater success in low-resource point-of-care diagnostics [11–15]. Although often low-cost, portable and easy to use, they have limited capacity to run multiplex, complex or a wide range of protocols and are often not as quantitative as traditional microfluidic assays except when paired with specific external readers [16]. Furthermore, due to inherent design limitations of capillary flow in a porous medium, paper microfluidics often cannot take advantage of droplet-based assays that are highly sensitive due to further reduction in associated fluid volumes and discrete and isolated nature of trapped fluid samples inside droplets. To address all the challenges listed above, the ideal technology would therefore have the capacity to (a) run complex, programmable, multiplex assays while being self-contained, (b) be capable of handling large fluid volumes in applications where the biological sample has few targeted events, (c) operate with both single phase and multiphase microfluidics and (d) would not require specialized training or any other external equipment.

Here we present a programmable multiplex microfluidic system based on punch card programming that is hand-crank powered, low-cost, robust, and can run complex biological and chemical protocols with limited chances of human-error. Moreover, our system is rugged, portable, hand-held (weighing approximately 100 grams and measuring approximately 2 inches in length, 1.5 inches wide and 1 inch high) and is self-contained. The system does not require any external pumps or other supportive equipment to run. Multiple protocols can be run in parallel (multiple assays on the same sample or single assay on multiple samples), manipulating fluids arbitrarily with nanoliter volume precision. Because the program is encoded in punch card tape, the protocols can be easily shared like baseball cards to repeat or modify existing assays.

Our current implementation is inspired by punch card programming as historically applied in a wide range of applications beginning with control of textile looms [17], early computing [18] and music replay [19]. Punch cards enable the use of a single actuating platform to execute multiple programs resulting in implementation of complex instruction sets that can yield radically different outcomes by simply switching the punch card tape. Such a platform offers the flexibility of achieving multiple results without the need to redesign the system for new tasks. We have harnessed this approach and implemented it to manipulate fluids in a low-cost platform.

## Mode of Operation

**Fig 1:**
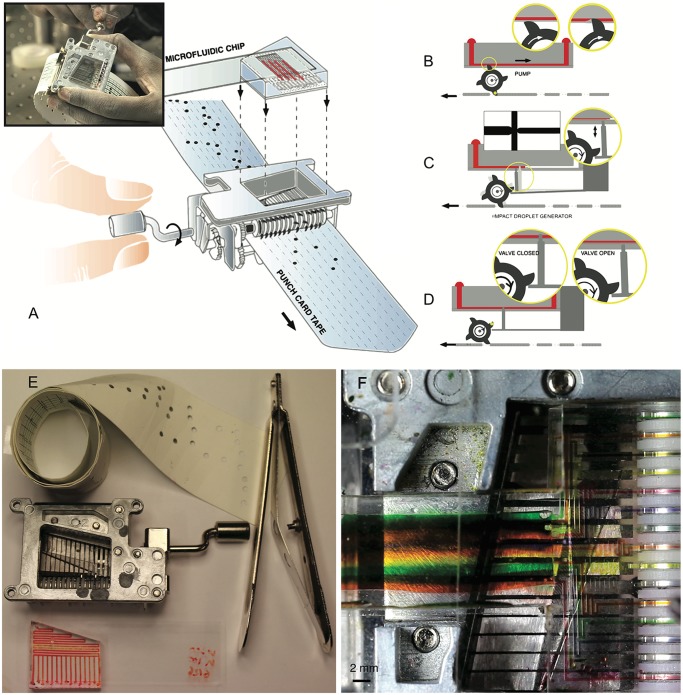
(A) Punch card programmable microfluidic system comprising of a paper punch card tape, plug-in microfluidic chip and a mechanical reader/actuator (inset depicts hand-crank powered device in action). The punched tape moves through the device while being read sequentially. (B, C, D) Schematic depiction of device operation including (B) pumping achieved by rotating gear teeth interacting with a collapsible channel, (C) on-demand droplet generator using an impact-based jet formation (D) normally-closed microfluidic valves based on cantilever pins pushed against a chip. (E) All components including paper tape, mechanical reader/actuator, microfluidic chip and a hole-puncher for encoding the paper tape. (F) Top-down micrograph of the device with 15 active channels filled with colored fluids.

Our system is comprised of a paper-based punch card tape, a polydimethylsiloxane (PDMS) based single layer microfluidic chip and a mechanical reader/actuator that couples fluidic channels to paper tape (Fig. 1*A–F*). The platform implements three key components required in general purpose microfluidic processors: embedded flow-controlled micro-pumps, normally-closed valves and a novel impact based droplet generator (Fig. 1*B–D*). The mechanical reader/actuator is powered by a hand crank (Fig. 1*A*, inset) and interfaces with the punch card tape to uniquely read the punched code and correspondingly execute pumping, valving and droplet generation in a microfluidic chip. Our modular design enables microfluidic chips to be inserted and removed from the device in a plug-and-play fashion.

Protocols are encoded, stored and executed using paper tapes (width 41 mm). Our current implementation consists of a series of 15 parallel lines on which holes (5mm diameter) can be punched to actuate 15 independent pumps, valves or droplet generators (see Fig. 1*F* and S2 Fig.). We demonstrate one-to-one correspondence between a hole and execution of pump/valve/droplet generator. The device has a bandwidth of 15 independent bits that can be set simultaneously. Other coding schemes can be easily implemented by mechanical re-configuration.

For the current reader/actuator implementation, we exploit the mechanism of a Kikkerland Music Box—a toy readily available in the market. Although many such mechanical music toys exist, we utilize the open architecture in the Kikkerland setup for quick prototyping. A completely 3D printed version of the reader/actuator was also implemented (see S1 Fig., inset) as an initial step in the rapid development of subsequent generations of our device. The reader/actuator consists of a gear train powered by a hand-crank coupled to a rotating rod that reels in the punch card tape with an approximate gear ratio of 1:6. To initialize the device, punch card tape is inserted in a slot comprised of thin metal sheets that act as guides toward two counter-rotating rods that are coupled to a series of gears. The plastic encasing of one of the rods provides additional friction enabling the turning rods to effectively reel in the punch card tape as the hand-crank is turned. The driven rod coupled to the gear train also consists of 15 gear discs that share the rod as an axle and have four asymmetric teeth positioned 90 degrees apart from each other (Fig. 1*B–D*). Plastic spacers between metal discs result in a 2 mm spacing between adjacent discs with gear teeth. The discs have the capacity to move independently and are only engaged when a punched hole appears in the paper tape. As the punch card tape is reeled in, the gear tooth that is closest to the hole eventually gets caught up in the hole and gets pushed in the direction of the actuation of punch card tape (+Y-axis). This action results in a rotation of the disc and therefore the other three gear teeth on the same disc also rotate in concert. For implementation of valves and droplet-generators, a secondary cantilever based array of pins is also coupled to the rotating gear train, enabling a single hole to actuate a vertical pin (Z-axis) to move up and down (see S1 Movie).

A microfluidic chip is coupled to the device in a plug-and-play fashion, held in place only by friction. Each chip consists of patterned single layer microfluidic channels on a thin membrane of PDMS (thickness 500 +/- 50 *μ*m) and couples directly to the gear train from the reader/actuator. The microfluidic channels are self-aligned to gear teeth and pins for fluidic pumping or valving with each actuation instance. We mold single layer microfluidic channels using standard soft-lithography with channel height of 50 *μ*m and width of 200 *μ*m. This thin film is bonded to a thicker slab of PDMS that provides mechanical support, inlet and outlet channels and means to couple the device to the reader/actuator. Next, we describe and demonstrate pumping, enhanced mixing, valving and on-demand droplet generation.

### Pumping

Integrated microfluidic pumps remove the dependence of a fluid-handling platform from external, bulky syringe pumps or pressure sources. Several implementations for microfluidic pumps have utilized the deformable nature of PDMS channels including pneumatic Quake-valve pumps [4], braille display based devices [20], finger-actuated flows [9], passive capillary pressure pumps [21] and external motors driving pins that actuate fluidic cavities [22]. While effective, they often require expensive external components (solenoid valves) and/or bulky pressure sources and do not always provide independent multiplexed control.

In our current platform, we have implemented 15 integrated and independently controlled micro-pumps. The net output flow-rate can be controlled as a function of actuation frequency and actuation height (Fig. 1*B* and *F*). Actuation frequency for a particular pump is dependent on the total number of punched holes on the paper tape engaged per unit time. The actuation height (*h*) is determined by how the microchip interfaces with the gear teeth. The power source for the device is a hand-crank.

**Fig 2:**
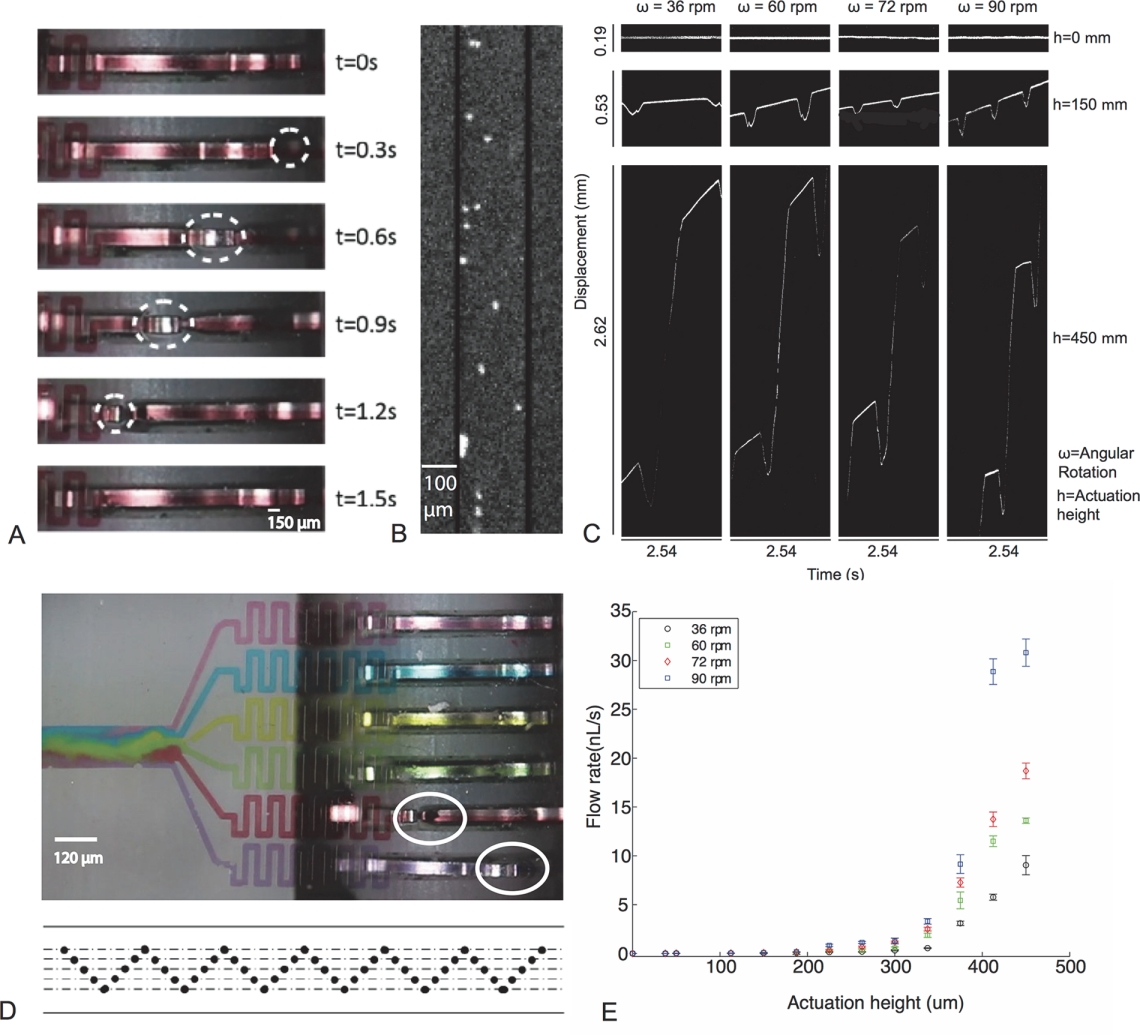
Punch card controlled integrated multiplexed microfluidic pumps. (A) A series of images of a single channel coupled to the rotating gear disc during one pumping cycle. (B) To characterize the flow pattern with each actuation, fluorescent polystyrene beads (2 *μ*m) were used in de-ionized water. (C) Pumping in each cycle revealed a characteristic asymmetric pulsatile oscillatory flow depicted above as a kymograph. The amplitude of directed unidirectional flow depends on actuation height (*h*) and the angular velocity (*ω*) from the hand-crank. (D) Top-down view of the microfluidic chip with simultaneous operation of six punch card controlled integrated micro-pumps. Net flow rate in a fluidic line is a function of exact pattern of punched hole (number of holes punched and spacing between the same, an example pattern depicted above). (E) Effective flow rate characterized as a function of *h* and *ω*, easily achieving typical values demonstrated by integrated micro-pumps.

The basic principle of the pump is illustrated in Fig. 1*B*, where a rotating disc couples a gear-tooth to a completely collapsible microfluidic channel. As the disc rotates, the squeeze contact point linearly translates the collapse position along the channel (Fig. 2*A*). The width of the channels (200 *μ*m) was further chosen to be less than the gear teeth width at the actuation area (~480 *μ*m wide) to ensure that the channel collapse is complete with each pumping cycle. The gear tooth engages with a given channel once for a single hole. With such a wide area of actuation for the pump, multiple channels can also be tied to the same gear teeth (single channel on the tape) further multiplexing pumping. To demonstrate operation of multiple pumps simultaneously, six independent pumps were operated with a “zig-zag” pattern of punched holes in a paper tape (Fig. 2*D*), resulting in continuous programmable operation of all the micro-pumps with desired flow rate set by frequency of holes in individual channels on the tape.

The vertical position of the microfluidic chip with respect to the reader/actuator determines the degree of collapse and its interaction with the microfluidic channel. Thus we can quantify the fluid flow in the channels with two simple parameters: height of the chip above the actuator (*h*) and angular velocity of the gear disc under rotation (*ω*). To characterize the pumping, fluid flow was imaged using red fluorescent microspheres (size 2 *μ*m) (Fig. 2*B*). Individual trajectories of the beads, depicted as kymographs reveal oscillatory dynamics of the fluid flow as a function of *h* and *ω* (see Fig. 2*C* and S4 Fig.). For the purpose of data collection, precise angular velocity (*ω*) was implemented using a motor driving the hand-crank. The oscillatory dynamics arise from the complete collapse and ensuing recoil of the channel as a result of the elasticity of PDMS, leading to a forward and backward flow. The asymmetry in forward and back flow is introduced due to preferential movement of the channel collapse point in the forward direction. The net effect of the back flow is limited with increasing actuation height (Fig. *2E*). For angular velocity (*ω*) of 1.5 rotations per second and displacement height *h* = 450 *μ*m, a net flow rate of ~30 nL/s can be easily accomplished. Below the critical value *h* = 50 *μ*m, no net forward flow is observed in the channels due to insufficient coupling of the rotating gear disc with the PDMS channel. With increasing actuation height, the arc across which total channel collapse occurs with each actuation increases leading to increased fluid flow with each rotational stroke (Fig. 2*E*).

To characterize the robustness and determine wear and tear of the device, a long-time experiment was run at an angular velocity of 1.5 rotations per second and actuation height h = 400 *μ*m (net flow rate of ~10 nL/s). After 100 hours of continuous operation (~ half a million cycles), the channels were still functional although worn down from the friction between the gear teeth and PDMS. To solve this problem of wear-and-tear over prolonged use, we developed a simple solution of applying Scotch tape on the exterior parts of the PDMS (at the point where the chip interfaces with the gear teeth). We demonstrate that this wear can be completely eliminated by this very simple solution. A test run for 100 hours of continuous operation at an angular velocity of 1.5 rotations per second (equivalent to 540,000 repeated cycles) at an actuation height (~450 *μ*m, equivalent to net flow rate ~30 nL/s) displays no visible sign of wear and tear on the PDMS.

### Enhanced fluid mixing

Fluid mixing is a significant challenge in integrated microfluidic devices due to lack of fluid inertia at low Reynolds numbers. Complex micro structures such as herringbone geometry [23] have been utilized to implement mixing in single-phase flow. These work by extending the interfacial boundary between two miscible fluids, thus increasing the effectiveness of diffusion across this interface thus enhancing mixing. Here, we present a simple strategy for fluid mixing on our platform that requires no special fabrication steps and is based on a simple pattern of encoded holes on a punch card tape. Because mixing is induced in a programmed manner, it is possible to effectively turn mixing ON and OFF in our devices.

**Fig 3:**
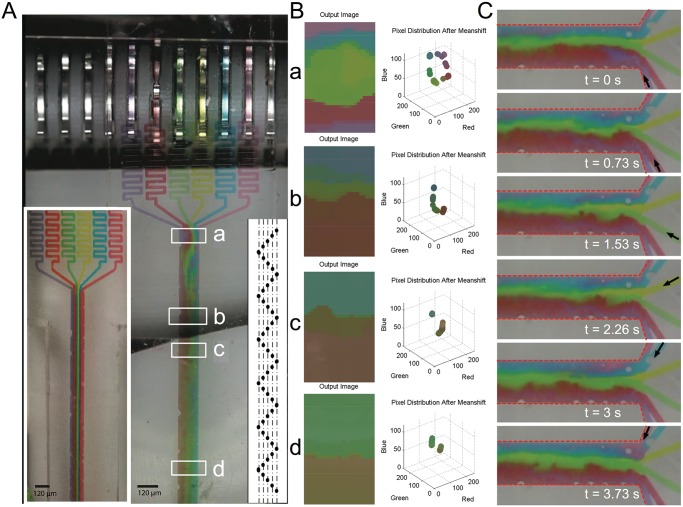
Enhanced mixing is achieved using a zig-zag pattern of punched holes. (A) Photomicrograph of six punch card controlled pumps driven by a zig-zag pattern (right inset). Left inset depicts the same device run through a traditional syringe pump (at the same flow rate) to highlight the striking difference in fluid mixing at the end of the channel (200 *μ*m wide). (B) Mixing is quantified by mean-shift clustering approach (see methods for details) comparing four regions in the micro-channel marked a, b, c, d along the outflow. Six identified clusters merge into two. (C) Photomicrographs from video data reveal the mechanism for mixing. Pulsatile nature of flow induces increased folding of neighboring flow lines (and hence net interface length) thus enhancing diffusion and mixing.

By exploiting the precise control of relative actuation time (controlled by distance between punched holes) of each of the pumps operating adjacent channels, we implement a simple mixing strategy in our devices (Fig. 3*A*). As a demonstration, we mixed six different water-based fluids simultaneously in a single output channel. The fluid was pumped through the device using the punch card tape at a flow rate of ~100 nL/s with an effective Reynolds number of 0.2. Even at such low Reynolds numbers, efficient mixing was achieved within 16 mm downstream of the inlet channels. Since all pumps can be independently controlled by 15-channel punch card tape, the diffusion boundary between nearby fluid streams (for instance, red and green streamlines) can be folded significantly by offsetting the moment of flow injection. As an example, we demonstrate mixing using a “zig-zag” pattern of punched holes (Fig. 3*A*, right inset). The time-varying flow rate induces interfacial boundaries amongst neighboring streamlines to significantly fold, enhancing diffusion (as depicted in the time series in Fig. 3*C*). In addition to the “zig-zig” pattern, two other punch tape patterns were implemented leading to different observed mixing levels (see S6 Fig.). Various other patterns can be explored to study mixing efficiencies.

To confirm that the folding only arises due to offset and pulsatile nature of our punch card controlled integrated micro pumps, we ran an equivalent experiment using a traditional syringe pump replicating the exact flow rate and flow geometry, as generated by our integrated micro-pumps. The streamlines did not mix and the six colored fluids remained effectively separated (Fig. 3, left inset). We further quantify the extent and speed of mixing in the six fluid streamlines from the six colored fluids pumped using a mean shift clustering algorithm [24–26] implemented on images taken along different points (white boxes marked a, b, c, d) along the channel (labeled with food color in water). See supplementary material (S1 File) and S3 Fig. for detailed implementation of the algorithm. Prior to mixing, six clusters corresponding to the six fluid colors were identified (Fig. 3*B*) and as samples were analyzed along the fluid channel, the number finally reduced to two clusters within a travel distance of 14 mm along the outlet channel (Fig. 3*E*).

### Valves

Successful and arbitrary manipulation of fluids to run a wide range of chemical and biological assays requires the use of micro-valves to enable programmable spatiotemporal control of fluid flow. Combined with integrated pumps, valves can easily facilitate complex flow control strategies, as have been previously demonstrated [4, 5] in multi-layer microfluidic valve structures. Numerous large-scale integration architectures based on valving schemes have also been described, including precise design rules for operation [27]. Most implementations of dynamic programmable valves involve external electrical solenoids, external pressure sources and expensive electronic control. The need for external control systems often limits the impact that valve based microfluidics could have in resource-poor settings.

**Fig 4:**
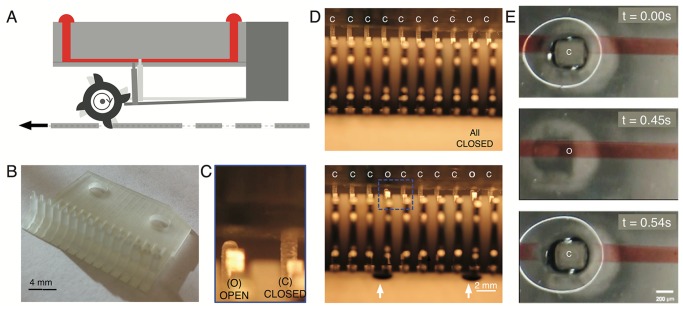
Integrated punch card controlled normally-closed valves. (A) Schematic of normally-closed valves depicting mechanism of operation. (B) 3D printed cantilever beam array with spaced pins (2 mm apart), utilized for implementing 15 independent normally-closed valves. (C, D) Micrograph from video of ten normally-closed valves under operation (side view), all of which are independently actuated based on the punch card tape. (E) Normally-closed valve in action, at a single instance of opening and closing depicting the time duration for a single cycle (0.54 seconds). The image depicts the entire region of PDMS deformation with a completely collapsed channel.

We successfully implemented multiple, independently controlled, punch card programmable micro-valves (Fig. 4). The valving mechanism comprises of independent pins (~400 *μ*m diameter) projecting perpendicularly (along the Z-axis) from a series of cantilevers that are attached to the base plate of the reader/actuator (Fig. 4*B*). In the default setting, the valve pins are passively aligned to the microfluidic channels pushing against the PDMS and thus collapsing the channel at the point of contact (OFF setting, Fig. 4*D* and *E*). During actuation, the gear train teeth pluck the cantilever beams, opening the channel due to the downward and forward motion of the valve pin. The channel is closed due to elastic recoil of the cantilever and pressure from the pin (Fig. 4*C* and *D*). A single cycle of a valve can be implemented in approximately 0.5 s (Fig. 4*D*). The implementation described above needs to be actuated to be in the open state (closed being the default state) to let fluid flow. A reverse valve that is open in the default mode can be implemented by simply reversing the leverage point of the cantilever.

As a demonstration, we implemented simultaneous valving and pumping in our device. This capability was made possible by simply shortening the valve pin. The microfluidic chip was then lowered to a height that would allow for integrated pumping (Fig. 1*B*) and valving (Fig. 1*C*) with each hole on the punch card tape. The coupled action allowed the valve to be open only when the fluid was being pumped and closed as soon as this action was complete. Because 15 independent punch card tape channels are available on the current implementation, the same number of independent valves and pumps can be operated simultaneously.

### On-demand droplet generator

Droplet microfluidics provides a platform for high-throughput and highly sensitive assays [28–30]. Although bulk generation of monodisperse droplets has been routinely demonstrated, current on-demand droplet generation [31] and control [32–34] in microfluidic channels requires complex external controllers and imaging based feedback. Here we demonstrate a new on-demand droplet generator programmed using our punch card ticker tape utilizing impact dynamics of a cantilever based pin on a soft substrate for single droplet dispensing.

**Fig 5:**
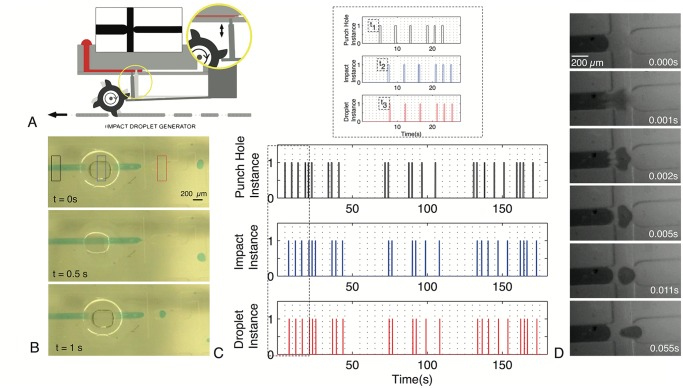
Demonstration of an impact based on-demand droplet generator. (A) Schematic of droplet generator operation. A cantilever beam plucked by a gear tooth induces a pin to impact the channel, in correspondence to punched holes. (B) Photomicrographs of on-demand impact droplet generator at a flow-focusing geometry. Water droplets (green food coloring in DI water) were dispensed in mineral oil (viscosity 15cP, surfactant 2%v/v Tween 20). (C) Timing control between arrival of punched hole, corresponding impact of the pin and the resultant single droplet formation (inset depicts magnified view with time delay between the three events). (D) High-speed imaging of droplet generation reveals an impact jet that forms in the first 2ms of the impact. The jet is quickly destabilized with formation of a narrow thread that breaks into a single droplet. Sequence of images depicts the entire operation ending with a single droplet formation over a short period of only 55ms.

The droplet generator presented here relies on the impact dynamics of a small pin-head on a compliant microfluidic channel to generate a fluid jet in a microchannel. The jet destabilizes very quickly (in the first 2 ms) resulting in a single mono-disperse droplet dispensed in surrounding carrier fluid of higher viscosity (See *S3* Movie and Fig. 5*A, B* and *D*). Unlike dynamic droplet generators that rely on co-axial continuous flow [35], resulting in a stream of droplets, only a transient pressure pulse (generated by pin impact triggered from a punched hole) is utilized in our system.

The impact droplet generation mechanism was implemented by using the same actuation pins (mounted on cantilever beams) as used in valving (Fig. 4*B*). The stiffness of the cantilever beam can be further tuned to regulate the time dynamics and remove any associated ringing effects upon impact. The impact pin height was tuned so as to only allow the cantilever mounted vertical pins to interface with the channel with no interference from associated gear teeth. The impact pins had a default “ON” position that led to complete channel collapse sealing the micro-channel. A flow-focusing geometry with a capillary valve was used (Fig. 5*A*, inset) for on-demand droplet generation. The carrier fluid (mineral oil, viscosity 15cP) has a flow rate of ~0.5 ml/min to move the droplets along the channel and away from the junction geometry and play no critical role in droplet generation.

One-to-one correspondence is achieved between a single hole in the punch card tape and the impact of the pin leading to a single droplet formation in a micro-channel (Fig. *5C*, *S3* Movie). Although the punch card holes only arrive every few seconds, the actual drop formation is nearly instantaneous (completed in ~50 ms). We filmed this process using a high-speed camera revealing the unusual shape evolution of the jet post-formation and its subsequent collapse (depicted in Fig. 5*D*, *S4* Movie). Dispensing high-speed jets of water in a higher viscosity mineral oil solution (viscosity, ~15 cP at room temperature), results in unusual jet dynamics that are strikingly different from jetting regimes seen in coaxial flows or droplet jets dispensed in air [36].

**Fig 6:**
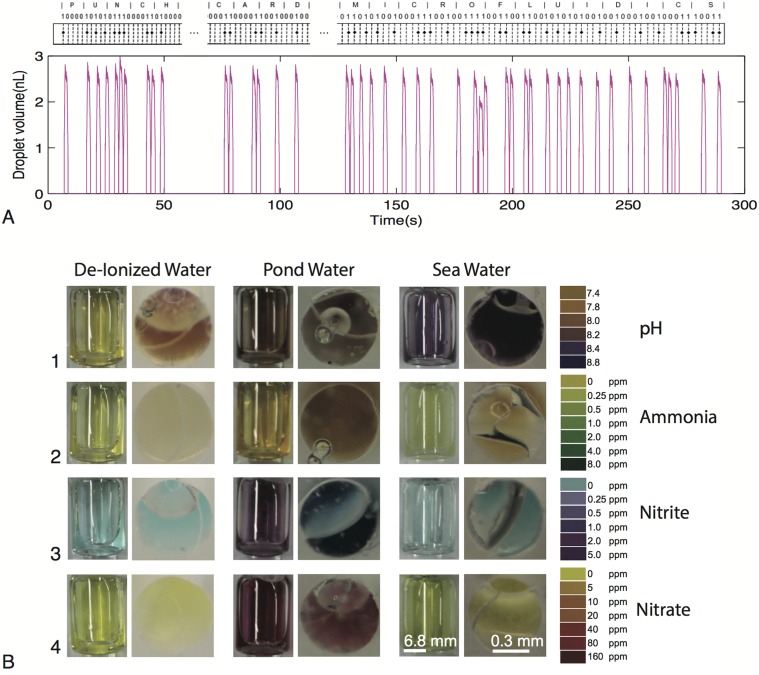
(A) One-to-one mapping demonstrated for the punched code and sequence of droplets generated. A binary code was implemented (every alphabet assigned a 5 bit code, example “U” = 10101) spelling “PUNCHCARD MICROFLUIDICS”. The presence of a hole in punch card tape (top) results in a single droplet representing a binary value of “1” while the absence of a hole results in no droplet representing a binary value of “0”. Vertical height of each plug represents droplet volume, which is approximately 2.5 nL. (B) Results of a qualitative colorimetric assay to test pH, ammonia, nitrates and nitrites in three samples: deionized water, pond water and seawater. Photographs compare bulk reactions (left image) with microfluidic outlet (right image); (1) pH readings with deionized water having a pH of 7.8, pond water at 8.2 and seawater at 8.8, (2) Ammonia levels in parts per million with deionized water at 0 ppm, pond water at 0.25 ppm and seawater at 0 ppm, (3) Nitrite levels with the deionized and sea water samples showing none and pond water at 2 ppm, (4) Nitrate levels in the water samples with pond water at 160 ppm and the deionized water and sea water samples having none.

To further demonstrate the robustness of our on-demand generator, we produce single droplets (volume ~2.5 nL) of water in mineral oil, whose timing is programmed by a long punch card code, thus establishing one-to-one mapping. We implemented a binary code consisting of 27 characters (alphabets and space bar) to write out “PUNCHCARD MICROFLUIDICS” using droplets generated by the impact droplet generation method (Fig. 6*A*). Five bits were used to encode each letter of the alphabet, and seven bits (all of which were zeros) were used to encode the space between words. The coding system followed the binary system such that the letter A was represented by “00001”, B = “00010”, C = “00011”… and so on. The code was programmed into the paper tape by punching holes whenever the high signal (“1”) was required. The “0” signal was represented by absence of punched holes, leading to no droplets being generated. In the results displayed on Fig. 6A, there is an apparent error on the letter “O” for the word “MICROFLUIDICS” that arose at the transition point between two punch card tapes joined together by Scotch tape that obscured a punched hole.

### Multiplex assay

We have presented a platform for general-purpose control of microfluidic systems using punch card ticker tapes capable of multiplex assays. Next we implemented a simple qualitative colorimetric assay to simultaneously test for pH, ammonia, nitrates and nitrites in three water samples collected: deionized water, pond water and seawater (Fig. 6*B*). Reagents from the API Colorimetric Water Test Kit (Aquarium Fish care, see methods section for details) were used to perform the assay. Fig. 6*B* reveals side-by-side results from the colorimetric test comparing a macro scale implementation to the output from our microfluidic outlet channels. From the colorimetric results, we were able to qualitatively demonstrate differences in pH, ammonia, nitrates and nitrites, across the three water samples.

## Discussion

Here, we have demonstrated a general platform with integrated microfluidic pumps, valves, mixers and on-demand droplet generators, with programmable control based on a paper punch card tape. No other external control elements are required. The current device demonstrates 15 independent programmable channels. This is not a fundamental limitation and can be increased by simply choosing higher density line spacing for gear discs or a wider punch card tape. The number of control valves can be increased further by multiplexing individual lines with blind channels filled with ionic fluids [20]. Wear and tear on the device over prolonged use was simply mitigated by using Scotch tape applied on the interface between the gear teeth and the PDMS (demonstrated with continuous operation for approximately half a million cycles, for 100 hours).

A reliable supply chain of reagents required for biological and chemical assays is difficult to maintain in remote resource-constrained settings. Further, the manipulation and storage of reagents currently use containers that are often expensive and can be difficult to dispose of [37]. To address some of these challenges, a recent paper by Bwambok *et al*. [38] demonstrated the use of bubble wrap pouches to store reagents and samples in liquid form and performing assays on them. We envision embedding dry reagents (for instance through lyophilization or the encapsulation using pullulan extract [39]) and/or wet reagents in both the punch card tape and/or the microfluidic devices for increased stability and shelf life. This would provide a single means of providing all the necessary reagents, tools and the protocol to run a given assay simplifying shipping logistics. As next steps, we will encapsulate reagents in the paper punch card medium that will allow for rupture on one side and will interface with the microfluidic chip on the other. An example of such packaging includes medical tablet blister packs, a process already compatible with roll-to-roll manufacturing. This functionality will further minimize reagents used, enable running of multiple complex assays with minimal training and no external handling of expensive reagents. The final result could also be deposited back on the paper tape as a repository for further analysis or record keeping.

Here we have demonstrated design principles behind a general-purpose punch card microfluidic device. Next, we will design new features in the mechanical reader/actuator including higher channel bandwidth (by simply reducing the spacing or increasing the length of the gear train) and developing a punch card programming language specifically targeting chemical assay. With easy to replicate components and portability, we intend to provide design specifications and rules to the broad global community to build new assays (akin to computer applications, Apps, written for smart phones), focusing on medical diagnostics, environmental monitoring and education.

## Conclusion

Combining punch card tapes with microfluidics provides a novel, scalable and inexpensive yet robust means to enable multiplex, general-purpose control of both single phase and droplet based microfluidic device. The platform is simple to use and its plug and play nature makes it accessible to both untrained health workers in the field and young children in classrooms. A given assay can be replicated simply by using an old punched tape, encouraging sharing of protocols, amongst users anywhere in the world. Since the mechanical components used here are based on a pre-existing toy, mass manufacturing for scale-up is self-evident. We believe that combining the capability to program general purpose microfluidic systems using punch card tapes and utilizing a hand-crank based power source can bring a broad range of capabilities outside of lab settings and into real-world field conditions. This makes future diagnostic instruments built on the presented platform truly portable. We envision that with our approach, hands-on chemistry and precise biological material manipulation can be brought into the hands of a wide range of new users in educational and resource-poor settings, enabling citizen science for curiosity driven explorations.

## Materials and Methods

### Microfluidic chip design and fabrication

We fabricated our microfluidic chips using standard PDMS photolithography techniques [40]. The device was composed of two PDMS layers. The channel features were fabricated to have a height of 50 *μ*m and a width of 200 *μ*m. The channels were spaced 2 mm apart so that they could be aligned to the actuators. In addition, the number of actuated channels was no more than 15, the maximum limit of the current reader/actuator. See the supplementary materials (S1 File) for additional details.

### Actuator and punch card tape

For the current implementation of the actuator, we used a Kikkerland Music Box and modified it as required, including new gear teeth profiles and associated cantilever beams with vertical pins for valving and impact droplet generation (see S1 Fig.). Punch card tapes (41 mm wide) were made out of simple card stock paper with guidelines drawn for punching holes. No limit was imposed on the length of the punch card tape. For current experiments, cantilever based valve pins were designed using AutoDesk Inventor CAD software and fabricated by 3D printing using a multi-jet modeling ProJet 3500 HD printer (Fig. 4*B*). This component was then fastened onto the base plate of the punch card driver in a way that allowed for programmable actuation of the valving mechanism.

### Reagents Used

Mineral oil (Fisher Scientific 8042–47–5, 15cP at room temperature) was used as the carrier fluid in the on-demand droplet generation experiments. The fluids used in the single-phase experiments and for the droplets immersed in the carrier fluid were made using food coloring (Wilton 8 Icing colors set) mixed with deionized water at a 1:10 v/v ratio. 2% v/v Tween 20 was added as a surfactant for all experiments.

### Flow Velocity Characterization Experiments—Pumping

To characterize the pumping capabilities of our device, red fluorescent 2 μm monodisperse polysterene microbeads (Polysciences Catalog No. 18660–5) were added to deionized water at a concentration of approximately 10,000 microspheres/*μ*l. The solution was pumped using our device through a channel that was 200 *μ*m wide, 50 *μ*m deep and 2.5 cm long (Fig. 2*B*). The punch card tape used to drive the actuator had holes punched 1 cm apart along the entire length of the tape. The ends of the punch card tape were joined together using Scotch tape for a continuous loop. A motor (Maxon DC motor Catalog No. 166789, Maxon Gear Catalog No. 218418) was attached to the hand-crank handle for characterizing flow rate at different angular velocity. The microchip was lowered onto the gear teeth in increments of 12.5 *μ*m from when the microchip made contact with the gear teeth to 450 *μ*m. Four different angular rotations (36, 60, 72 and 90 rpm) were used for each actuation height tested. Angular velocity of drive wheels for the paper tape effectively varies actuation frequency of the gear teeth coupled to the pumps. We investigate pumping rates as a function of this rotation velocity. See supplementary materials (S1 File) for additional details.

### Water quality test

A colorimetric assay for testing various compounds in water was implemented using the API Water Test Kit (Aquarium Fish Care Catalog No. 347). Filtered pond water, sea water and deionized water were used as samples to be tested. The punch card code was designed to match the volumetric ratio between the reagents and the water sample to be tested according to the manufacturer’s test kit protocol. The code was a one-to-one mapping of punched hole to reagent fluid volume introduced into the sample water line. Through valving, the reagent was introduced into the sample chamber only upon actuation. A multiplex assay consisting of five different reagent lines was implemented to test the water samples simultaneously. Colorimetric readings were done at the channel outlet where the fluid column along the light path was longest due to the combination of the thick PDMS slab and the thin channel layer. Images presented were taken using a Canon EOS Rebel t4i DSLR camera.

## Supporting Information

S1 Fig Models of the device reader/actuator. CAD model of the reader/actuator. Inset (*top*) depicts a milled version of an array of pins on cantilevers for implementing impact droplet generation and valving on out platform. Inset (*bottom*) is of a functional 3D-printed version of the device coupling both the punch card tape and the PDMS chip. Implementation on a 3D printed platform enables easy sharing of design files across the globe.
(TIF)

S2 Fig Punch card tape schematic. Schematic of paper punch card tape with arbitrary code demonstrating design rules for encoding information in punched holes. A minimum of 1 cm spacing is permitted between two holes following each other on the same channel. The current tape allows for 15 consecutive coding lines that are set 2 mm apart.
(TIF)

S3 Fig Use of Mean-shift algorithm in determining mixing efficiency. (A) Input raw image from a specific location in a micro-channel prior to processing with the pixel distribution in 3D feature space represented by RGB values (Red, Green, Blue). Processed output image with associated mean shift pixel distribution. (B) Flow chart of mean-shift algorithm implementation for pixel distribution clusters for quantification of mixing.
(TIF)

S4 Fig Flow characterization using a kymograph of a single 2 micron fluorescent bead to determine the net forward flow rate of the punch card programmable pump. Due to pulsatile nature of the pump, the bead is clearly seen to move both forward and backward in a single cycle. Net flow rate is computed by accounting for net forward displacement over a single cycle. The reported values are averaged for multiple such cycles.
(TIF)

S5 Fig Image of device, corresponding logic map and sample schematic of the implementation of valves and pumps. 15 independent valves and pumps are demonstrated in the logic map. The sample schematic depicts an example of the use of the valves and pumps as determined by the microfluidic chip design.
(TIF)

S6 Fig Implementation of three different punch card tape patterns to pump single-phase fluids (food color in deionized water at 1% v/v) from six inlet channels to a common reservoir leading to the output. (A) Depicts simultaneous pumping by all six pumps with each instance (B) is of serial pumping using a zig-zag pattern (C) depicts a pattern of simultaneous pumping episodes in three channels at each instance. Each of the three channels in a set are separated from each other by a single adjacent channel belonging to another set. Two sets were implemented, simultaneously actuated while being offset from each other in a temporal fashion. These images reveal differences in the level of fluid mixing achieved by simply switching the punch card tape pattern.
(TIF)

S1 File Supplementary information on methods and materials. (DOCX)

S1 Movie Valve pins. The front view of the device in action wherein the punch card tape reels in towards the observer upon actuation. The image focus is on the valve pins that get actuated whenever there is a punched hole in the card. Presence of the holes results in gear teeth plucking the cantilever beams on which valve pins project perpendicularly. This action leads to a downward and forward motion of the valve pins. The net downward movement results in the opening of the valve for the duration of the actuation (~0.5 s). The capture and playback rate for the movie is 60 frames per second.
(MP4)

S2 Movie Enhanced mixing. Top-down view of the device in action. Six input channels having different food coloring solutions are directed to an open chamber where mixing occurs. The pulsatile nature of the actuation results in enhanced mixing due to increased folding of fluid layers. The capture and playback frame rate for the movie is 60 frames per second.
(MP4)

S3 Movie One-to-one mapping of droplet impact. Top-down view of our impact droplet generator. The actuating pin forms a circular indentation on the microchip made using polydimethylsiloxane (PDMS) on channel closure. Mineral oil is used as a carrier fluid in a flow-focusing geometry. The droplets are of food coloring in deionized water. Capillary pin geometry was implemented on the fluid line carrying the water-based solution, at the point of intersection with the mineral oil lines. With each punched hole, the actuator pin has a net downward force before springing back to its original position. The impact during this process results in the ejection of a fluid stream that forms a droplet and is carried downstream in the oil line. The capture and playback rate for the movie is 50 frames per second.
(MP4)

S4 Movie High speed video of droplet generation. Impact droplet generation in our device captured at 1,000 frames per second. The circular indentation is of the actuator pin. As was the case in S3 Movie, flow-focusing microfluidic geometry was implemented. However, in this case, unlike in S3 Movie, capillary valve geometry was not implemented on the channel carrying the water-based solution at the point where it intersects with the oil line. Mineral oil was used as the carrier fluid and deionized water with food coloring was used for the water-based droplets solution. The playback frame rate is 5 frames per second.
(MP4)
