# Supplementary figures and images for: Punch Card Programmable Microfluidics
Source: PLoS One. 2015 Mar 4;10(3):e0115993. doi: 10.1371/journal.pone.0115993 (PMC4349784; doi:10.1371/journal.pone.0115993)

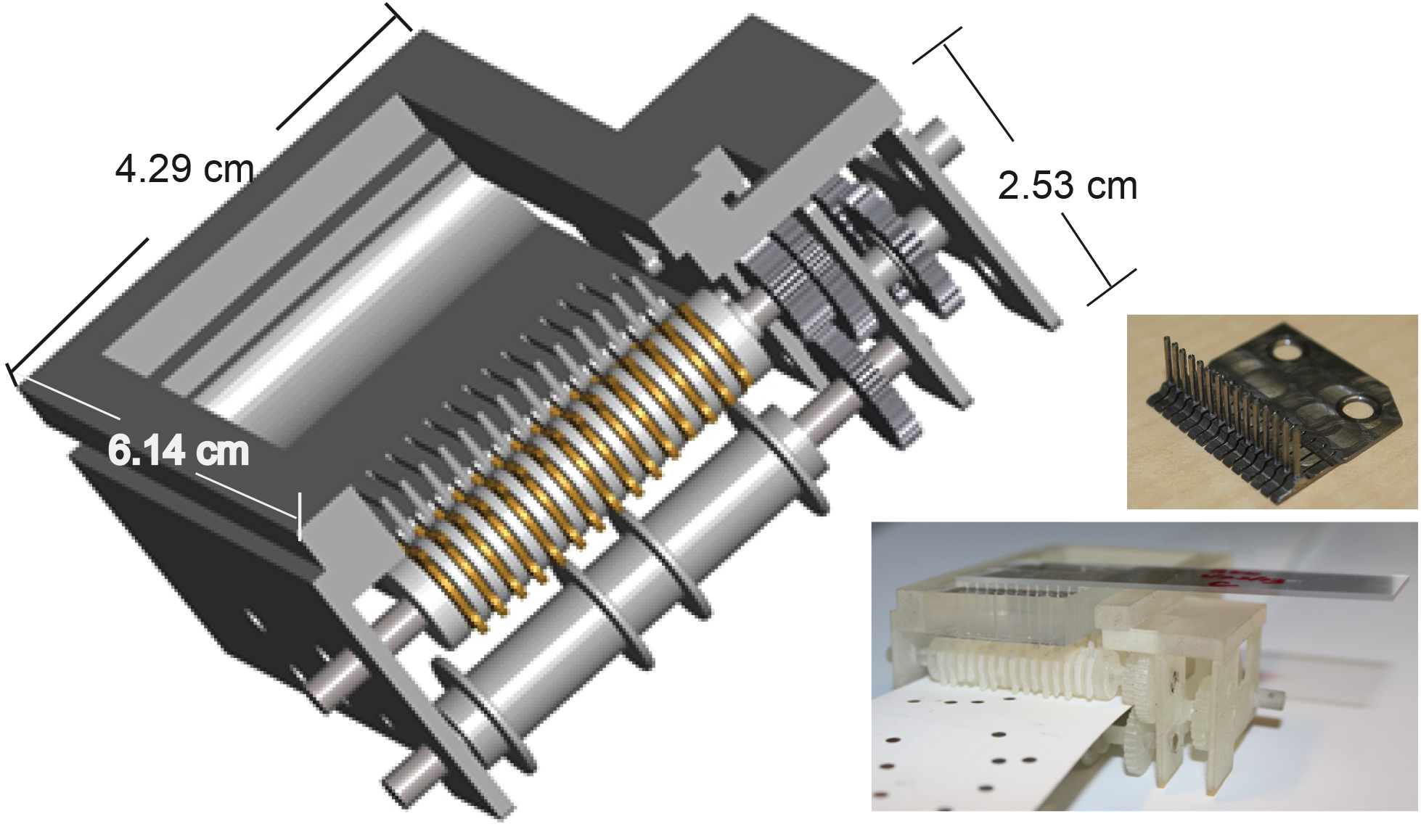

Supplement: S1 Fig — CAD model of the reader/actuator. Inset (top) depicts a milled version of an array of pins on cantilevers for implementing impact droplet generation and valving on out platform. Inset (bottom) is of a functional 3D-printed version of the device coupling both the punch card tape and the PDMS chip. Implementation on a 3D printed platform enables easy sharing of design files across the globe. (TIF) [file pone.0115993.s001.tif]

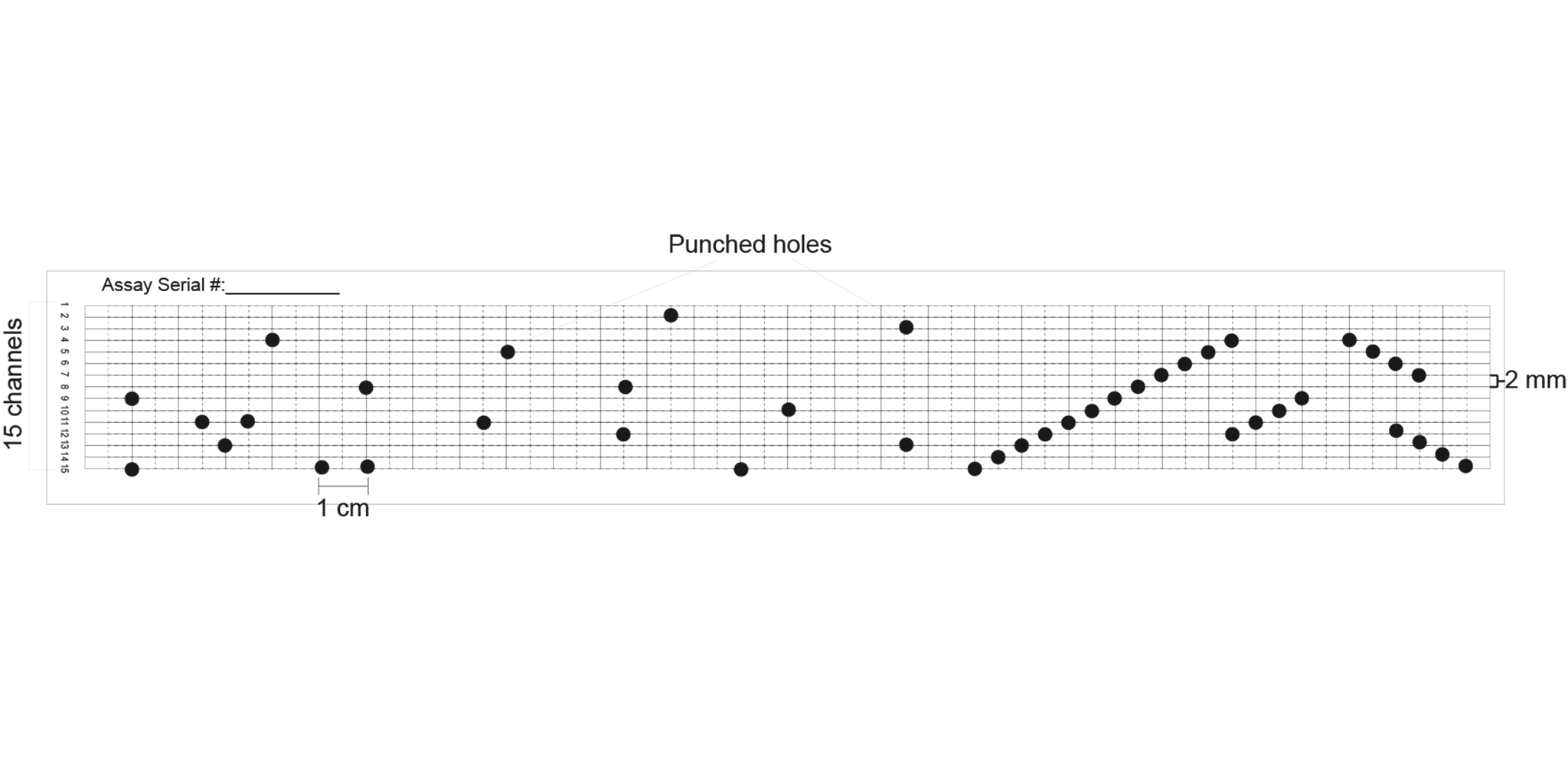

Supplement: S2 Fig — Schematic of paper punch card tape with arbitrary code demonstrating design rules for encoding information in punched holes. A minimum of 1 cm spacing is permitted between two holes following each other on the same channel. The current tape allows for 15 consecutive coding lines that are set 2 mm apart. (TIF) [file pone.0115993.s002.tif]

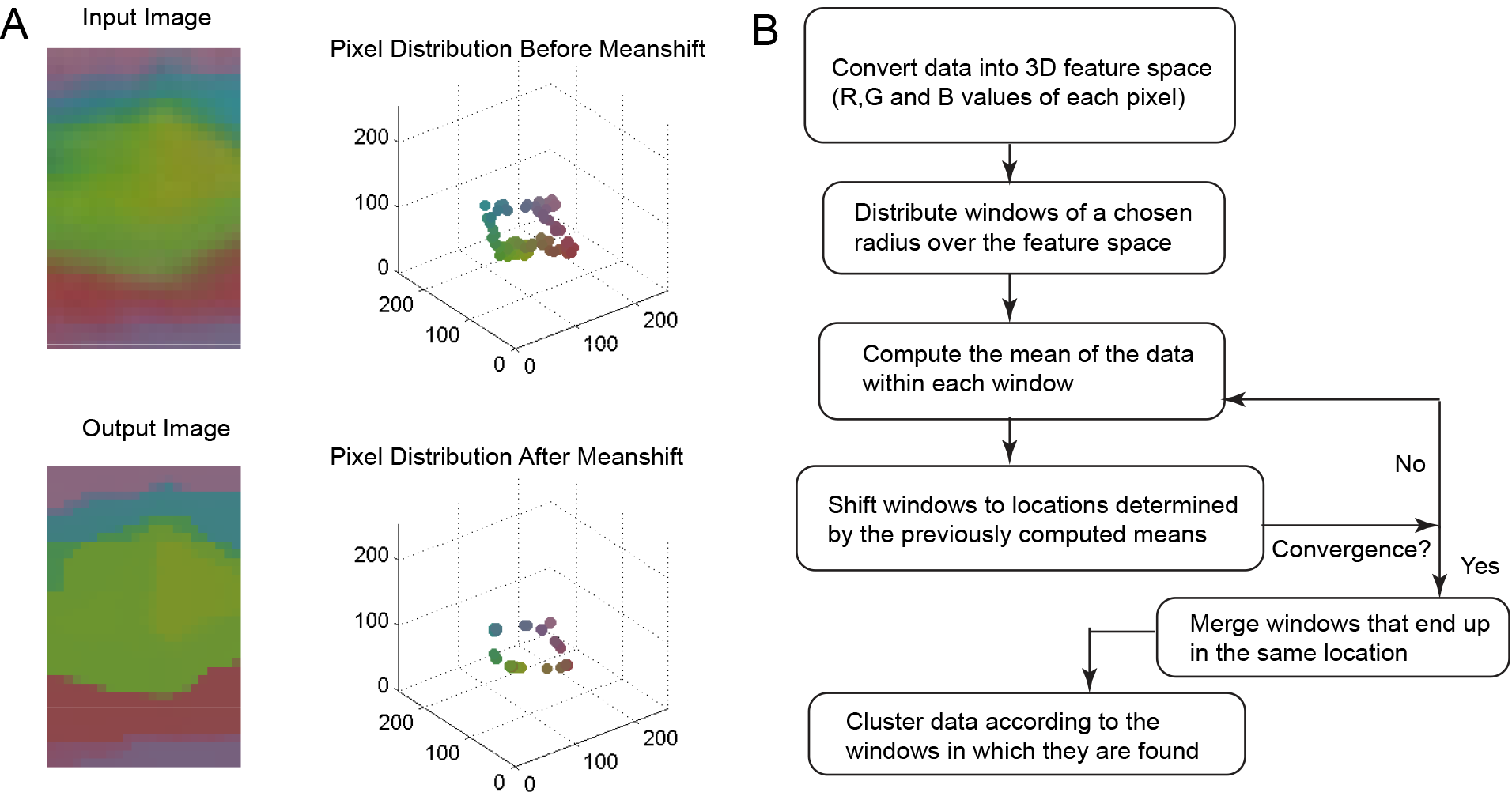

Supplement: S3 Fig — (A) Input raw image from a specific location in a micro-channel prior to processing with the pixel distribution in 3D feature space represented by RGB values (Red, Green, Blue). Processed output image with associated mean shift pixel distribution. (B) Flow chart of mean-shift algorithm implementation for pixel distribution clusters for quantification of mixing. (TIF) [file pone.0115993.s003.tif]

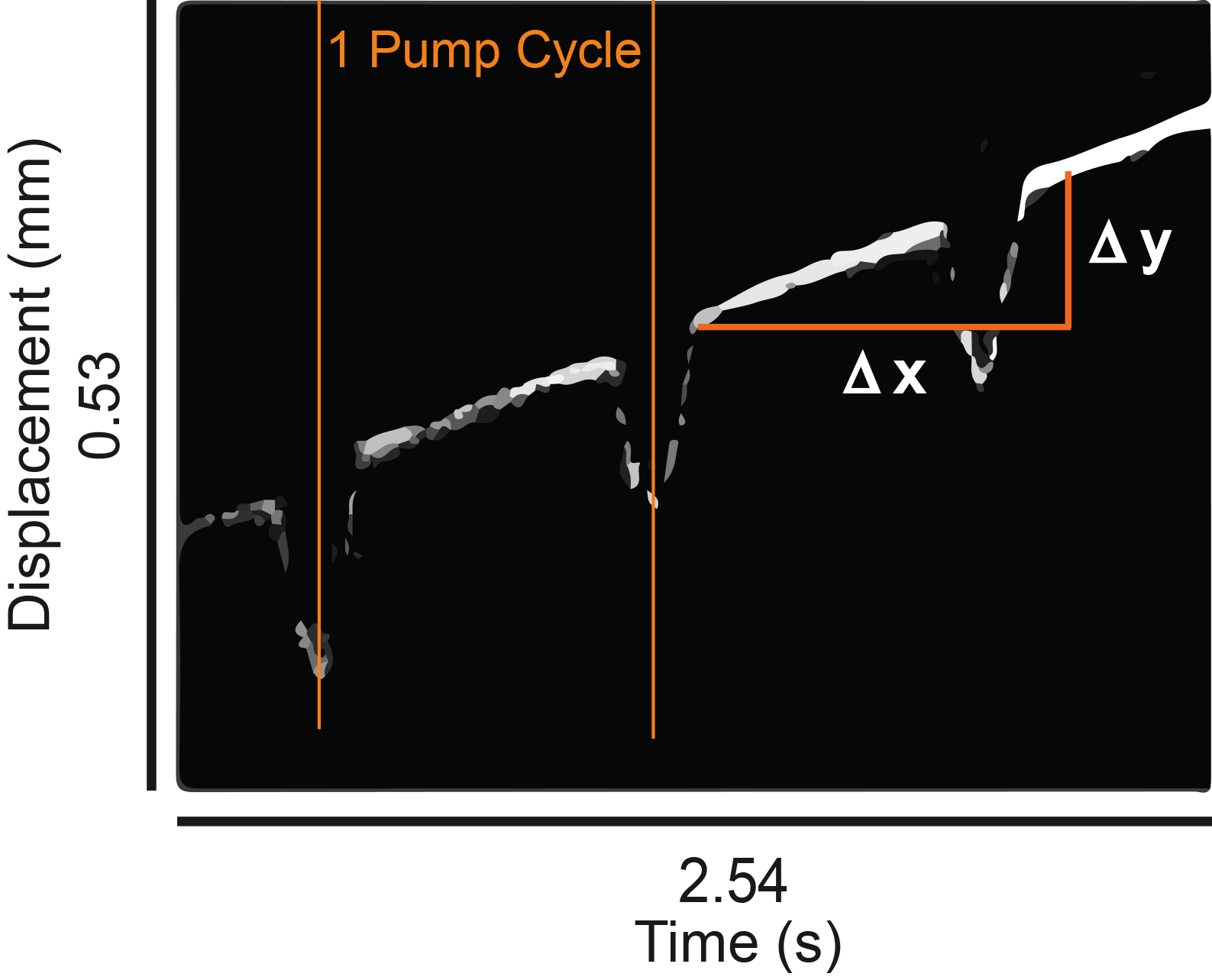

Supplement: S4 Fig — Due to pulsatile nature of the pump, the bead is clearly seen to move both forward and backward in a single cycle. Net flow rate is computed by accounting for net forward displacement over a single cycle. The reported values are averaged for multiple such cycles. (TIF) [file pone.0115993.s004.tif]

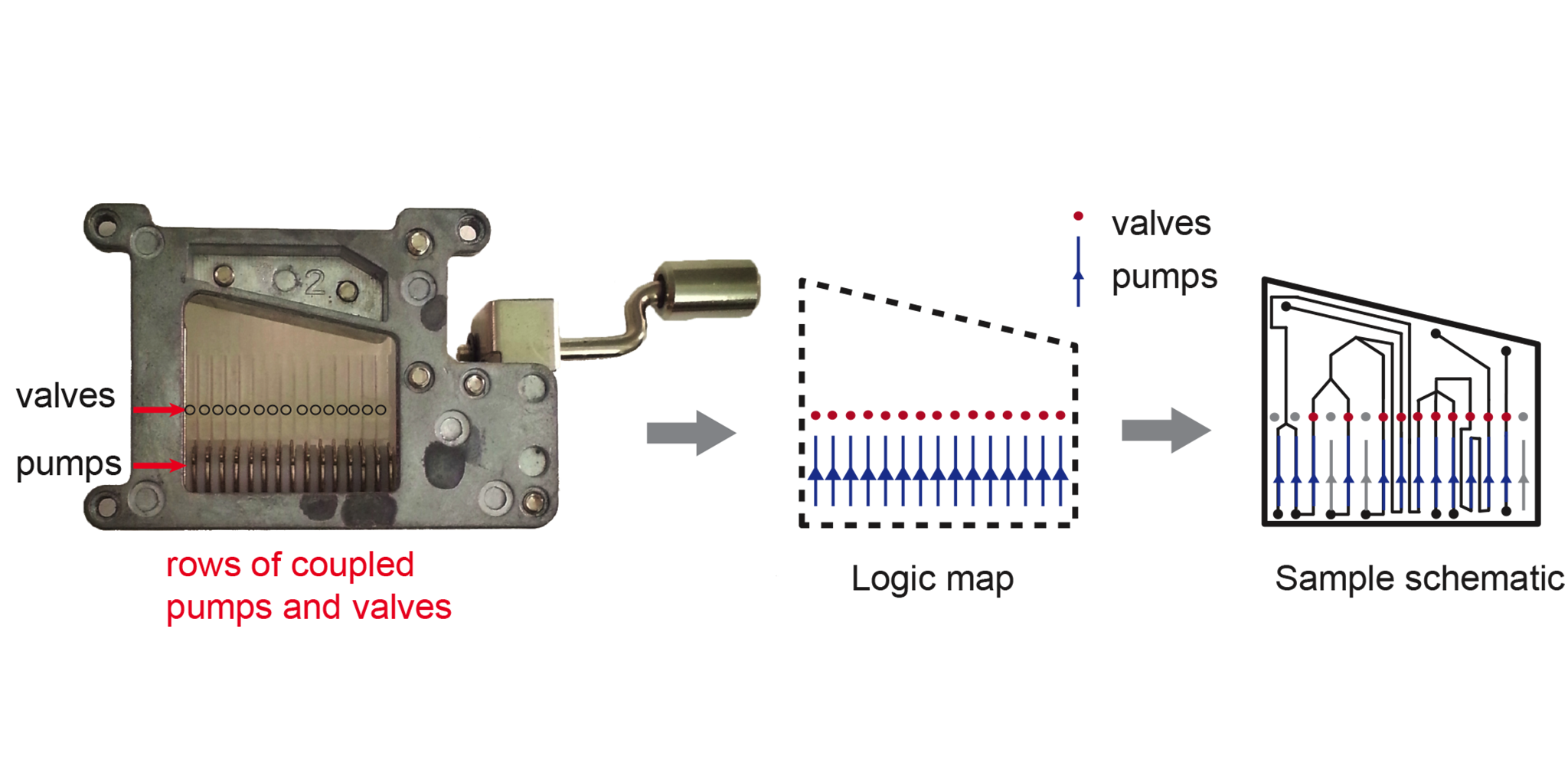

Supplement: S5 Fig — 15 independent valves and pumps are demonstrated in the logic map. The sample schematic depicts an example of the use of the valves and pumps as determined by the microfluidic chip design. (TIF) [file pone.0115993.s005.tif]

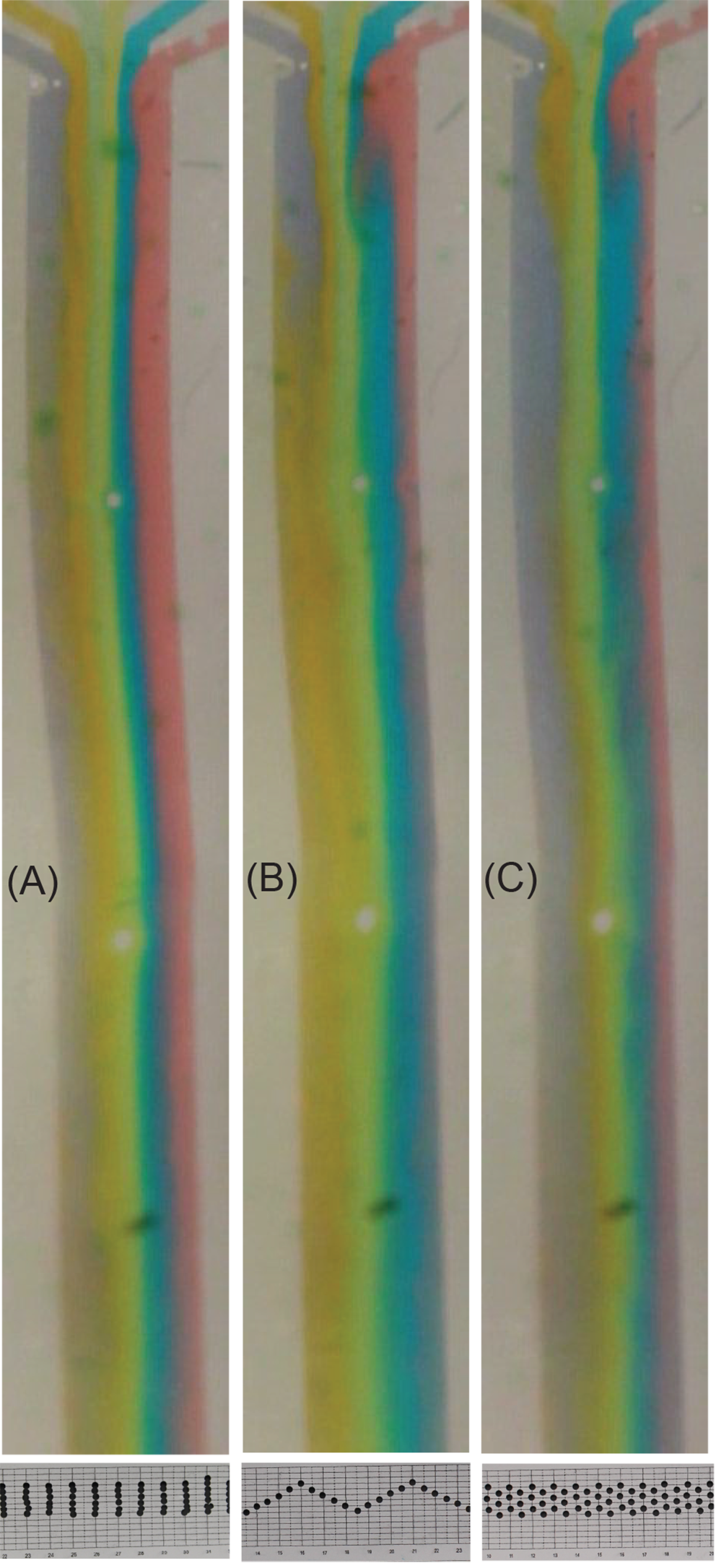

Supplement: S6 Fig — (food color in deionized water at 1% v/v) from six inlet channels to a common reservoir leading to the output. (A) Depicts simultaneous pumping by all six pumps with each instance (B) is of serial pumping using a zig-zag pattern (C) depicts a pattern of simultaneous pumping episodes in three channels at each instance. Each of the three channels in a set are separated from each other by a single adjacent channel belonging to another set. Two sets were implemented, simultaneously actuated while being offset from each other in a temporal fashion. These images reveal differences in the level of fluid mixing achieved by simply switching the punch card tape pattern. (TIF) [file pone.0115993.s006.tif]
