# Supplementary material for: Punch Card Programmable Microfluidics
Source: PLoS One. 2015 Mar 4;10(3):e0115993. doi: 10.1371/journal.pone.0115993 (PMC4349784; doi:10.1371/journal.pone.0115993)
Supplement: S1 File — (DOCX) [file pone.0115993.s007.docx]

**Supplementary Material**

**Punch Card Tape: punched hole spacing design rules**

The spacing between two adjacent tracks on the punch card paper tape was set to 2 mm, based on the geometry of the current mechanical reader/actuator (see Figure S2). This width helps avoid inadvertent actuation of neighboring channels. The spacing (along the tape, parallel to the actuator gear train) between holes sequentially passing through the device determines the relationship between the timing of actuation of corresponding channels. Based on the design of the actuator gear teeth (see Figure S1 for details of the mechanical design), it was determined that for each gear tooth, holes should be positioned at least 1 cm apart along the tape for unique actuation for each hole punched (see Figure S2). No limits are placed on the length of the program (governed by the length of the punch card tape). For specific applications, the same program (punched tape) can also be run in a circular configuration for continuous and repeated implementation.

**Mean-Shift Clustering Algorithm**

A pattern recognition algorithm known as the mean-shift algorithm [1-3] was used to quantify the level of mixing achieved using our device. With this technique, prior knowledge of the number of clusters is not required. For each chosen section, an image was taken and the red, green and blue (RGB) values of each pixel were taken as data points to be clustered. These RGB values were converted into the 3D feature space and windows of a certain radius were chosen arbitrarily. The mean was then computed within each window and the window shifted to a new location based on the mean. This process is repeated until there is convergence of windows. In addition each data point is shifted to the average of data points around it in an iterative fashion until there is convergence, and then clustering the data points around the closest convergence point of a particular window (neighborhood radius) (see Figure S3). Thus, pattern recognition was based on the mean shift approach applied to the analysis of individual images.

**Microfluidic chip design and fabrication**

The device was composed of two PDMS layers with different material properties bonded together. The first layer made of PDMS (polymer base to cross-linker ratio of 20:1) interfaced with the actuator and had the microfluidic channels. The second layer acted as an optically transparent firm-substrate against which the channels would be actuated while also being thick enough to ensure that the actuators interact with the micro channels effectively. This second layer was also made using a PDMS pre-polymer, mixed at a base to cross-linker ratio of 5:1. The layer with the channels measured approximately 500*µ*m while the thick layer measured approximately 7 $\mathrm{mm}$). The PDMS mold for the microfluidic channels was made using SU-8 and a negative photoresist mask.

The fabricated mold was used to form the channels using PDMS by casting the pre-polymer that had a base to cross-linker ratio of 20:1. In order to enhance the actuators’ effect on the microfluidic channels, the PDMS was cast by spin coating the layer of pre-polymer on the mold at 250 rpm for 30 s. The PDMS was then cured in an oven at 80°C for 30 minutes to make the layer that would be used sacrificially for the making of inlet and outlet holes punched through the second layer. This second layer with the base to cross-linker ratio of 5:1 had no features and so the pre-polymer mix was poured in a petri-dish and cured in an oven at 80°C for 30 minutes. When the time elapsed, the layer with channel features was peeled and placed on top of the thick layer. Inlet and outlet holes were then punched through both layers. Another casting iteration for the layer with the microfluidic channels was then done with pre-polymer being cured in an oven at 80°C for 30 minutes and used to replace the one that that was used sacrificially for the making of inlet and outlet holes. The two layers were then cured in an oven at 80°C for a minimum of four hours to ensure that they completely cured and bonded to each other. The cured and bonded PDMS device was then cut to shape and a glass slide was plasma bonded on the side that did not interface with the actuator. The glass slide only served as a handle for characterization purposes where the microfluidic device was raised or lowered as desired.

**Water quality test**

The test kit had reagents for testing pH from 6-7.6, high-range pH from 7.4 – 8.8, ammonia (up to 8 mg/dL), nitrites (up to 5 mg/dL) and nitrates (up to 160 mg/dL). The volumetric ratio of reagents to samples varied with the compound being tested.
**A. Bulk solution test protocol**
**i. pH bulk solution test protocol:** For the pH test, 5 ml of the bulk sample solution was mixed with 150 µl of the reagent solution. The solution was then left to sit in room temperature for 5 minutes before the colorimetric reading was taken. The color observed was compared against a color chart that corresponded to various pH values (Figure 5).
**ii. Ammonia bulk solution test protocol**: The ammonia test entailed the use of two reagents in 5 ml of the bulk sample solution. 240 µl of each of the two solutions was added to 5 ml of the sample and mixed together. The mixture was then left to sit in room temperature for five minutes before a colorimetric reading was done by comparing the color of the result against a chart with different ammonia concentrations (Figure 5).
**iii. Nitrite bulk solution test protocol.** In the nitrite test, 150 µl of the reagent solution was added to 5 ml of the sample to be tested and mixed. The resulting mixture was then left at room temperature for 5 minutes before the reading was made.
Nitrate bulk solution test protocol: The nitrate test required two reagent solutions to be added to the sample to be tested. 300 µl of each of the two reagents were added to 5ml of the sample and mixed together. The colorimetric result of the resulting solution was then read five minutes later.

B. Microfluidic chip implementation protocol

Each reagent test was carried out in a separate water line each having a volume 1.125 µl. For testing water pH, three fluid plugs each being 15 nL of fluid, generated by the punch card programmable platform were introduced into the water line. Five fluid plugs of the reagent were used for the high-range pH test and the nitrite test. Here a plug is defined as an actuation instance where the reagent is injected into the water solution. Each instance arises from a single punched hole. This translates to a volume of approximately 15 nL. The Ammonia test required 16 fluid plugs while the Nitrate test required 20 fluid plugs of the reagent being introduced into the sample water to be tested. The solutions were then incubated for five minutes before the color readout was done.

References

1. Comaniciu D & Meer P (2002) Mean shift: a robust approach toward feature space analysis. *Pattern Analysis and Machine Intelligence, IEEE Transactions* 24(5):603-619.
2. Fukunaga K & Hostetler L (1975) The estimation of the gradient of a density function, with applications in pattern recognition. *Information Theory, IEEE Transactions* 21(1):32-40.
3. Cheng Y (1995) Mean shift, mode seeking, and clustering. *Pattern Analysis and Machine Intelligence, IEEE Transactions* 17(8):790-799.
